# Life-Threatening Subglottic Stenosis of Granulomatosis with Polyangiitis: A Case Report

**DOI:** 10.3390/medicina57050423

**Published:** 2021-04-27

**Authors:** Jin An, Jae-Won Song

**Affiliations:** 1 Department of Pulmonary, Allergy and Critical Care Medicine, Kyung Hee University Hospital at Gangdong, College of Medicine, Kyung Hee University, Seoul 05278, Korea; anjin7487@gmail.com; 2 Department of Thoracic and Cardiovascular Surgery, Kyung Hee University Hospital at Gangdong, Kyung Hee University School of Medicine, Seoul 05278, Korea

**Keywords:** granulomatosis with polyangiits, wegener’s granulomatosis, subglottic stenosis, steroid, rituximab

## Abstract

Granulomatosis with polyangiitis (GPA) is an autoimmune disease characterized by necrotizing granulomatous inflammation. Subglottic stenosis, which is defined as narrowing of the airway below the vocal cords, has a frequency of 16–23% in GPA. Herein, we present the case of a 39-year-old woman with subglottic stenosis manifesting as life-threatening GPA, which was recurrent under systemic immunosuppressive therapy. The patient underwent an emergency tracheostomy, intratracheal intervention, such as carbon dioxide (CO_2_) laser surgery and intralesional steroid injection via laryngomicroscopic surgery, and laryngotracheal resection with remodeling. Severe subglottic stenosis treatment requires active intratracheal intervention, surgery, and systemic immunosuppressive therapy.

## 1. Introduction

Granulomatosis with polyangiitis (GPA) is an autoimmune systemic necrotizing vasculitis combined with granulomatous inflammation [1]. It may affect any organ system, but often involves the upper respiratory tract, lungs, and kidneys. Sinusitis and ulcerations of the nasal mucosa are common manifestations in the upper respiratory tract [2]. Subglottic stenosis (SGS), which is defined as narrowing of the airway below the vocal cords, has a frequency of 16–23% in GPA [2,3]. In patients with SGS, clinical symptoms such as stridor, hoarseness, and dyspnea require urgent evaluation and may be potentially life-threatening. Here, we present a clinical experience of GPA with life-threatening SGS, which improved with medical and surgical treatment.

## 2. Case Report

A 39-year-old Russian woman was admitted to the hospital because of severe, progressively worsening dyspnea and hoarseness for 1 month. She was diagnosed with chronic rhinosinusitis with mild ulceration of the nasal mucosa at the Russian medical clinic 2 months prior, with aggravated nasal blockage and blood-mixed nasal secretion despite symptomatic treatment. One month later, the patient complained of dyspnea and hoarseness without cough, sputum, fever, and chills. She was a never-smoker, with no other significant medical or operative history.

**Figure 1:**
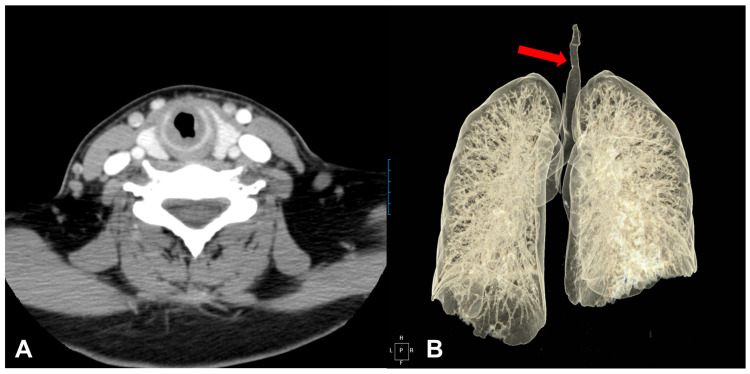
A chest computed tomography scan of the larynx and trachea revealed the subglottic stenosis with concentric circumferential thickening of the mucosa (**A**), and thickening of cricoid cartilage level to the medial level of the thyroid gland (**B**).

Upon examination, saddle nose and nasal mucosal edema were observed. Other abnormal findings, including skin lesions, were not observed. On auscultation, a severe wheezing sound without crackles was noted in both upper lung fields. Blood examination revealed elevated C-reactive protein (3.55 mg/dL) and erythrocyte sedimentation rate (66 mm/h). The full blood counts (including red cell count, white cell count, and hemoglobin), renal function, and urine analysis were within the normal range. Chest radiography revealed no remarkable findings, and paranasal sinus X-ray showed maxillary sinusitis with a mucosal thickening. On chest computed tomography (CT), there was no abnormal findings in the lung parenchyma; however, there was a diffuse thickening of the lower laryngeal and upper tracheal wall from the cricoid cartilage level to the medial level of the thyroid gland (Figure 1A,B).

**Figure 2:**
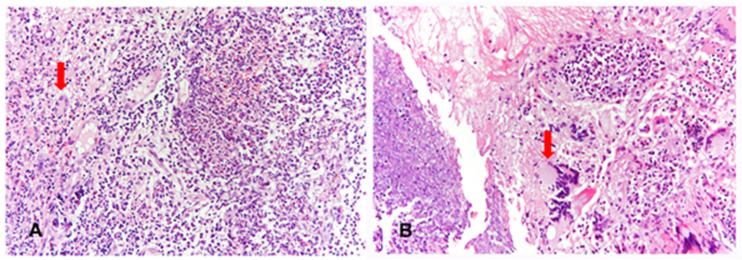
Histopathological findings of nasal mucosa at diagnosis (**A**) and bronchial mucosa at relapse (**B**). Nasal and bronchial mucosa show necrotizing granulomatous inflammation, consisting of basophilic necrosis, epithelioid histiocytes, and multinucleated giant cells (arrows). (hematoxylin and eosin statin, ×200).

We suspected GPA with upper respiratory tract manifestations, such as nasal discharge, saddle nose, and SGS. Serology showed positives values for c-antineutrophil cytoplasmic antibody (C-ANCA) (3+) and proteinase-3-ANCA (PR3-ANCA) (38.5 units). Anti-nuclear antibody, rheumatoid factor, and any viral markers revealed negative results. We planned to perform a biopsy of the nasal mucosa and examine bronchoscopy. Histological examination of the nasal mucosa revealed necrotizing granulomatous inflammation (Figure 2A). 

**Figure 3:**
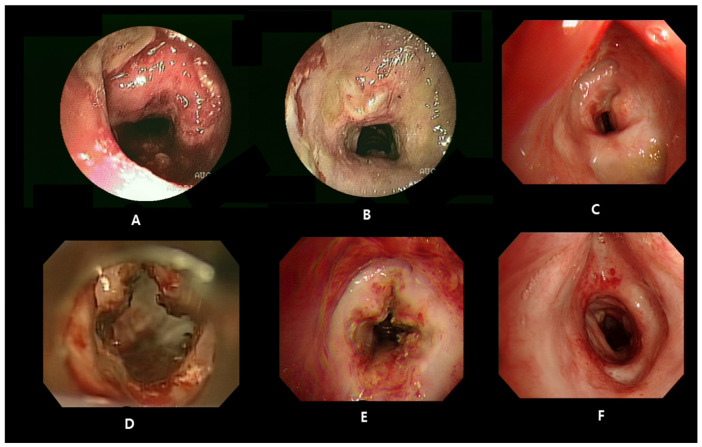
Initial bronchoscopy image showed severe inflammation with circumferential subglottic stenosis (SGS) (**A**). After intravenous steroid pulse therapy, SGS was improved (**B**), but restenosis was observed 5 months after discharge (**C**). Although CO_2_ laser surgery for luminal widening and intralesional steroid injection were performed (**D**), SGS recurred 1 week later (**E**). The patient underwent laryngotracheal resection with remodeling; SGS was improved (**F**) and she achieved decannulation.

Bronchoscopy revealed an irregular luminal narrowing of the subglottic area and upper trachea by 50% (Figure 3A). Finally, the patient was diagnosed with GPA with SGS based on the diagnostic criteria as defined by the American College of Rheumatology [4]. The patient received steroid pulse therapy (intravenous methylprednisolone, 1000 mg for 3 days) followed by oral prednisolone. Additional bronchoscopy was performed 5 and 12 days after steroid pulse therapy, and the upper trachea inflammation was improved (Figure 3B). On the follow-up chest CT scan, the diffuse thickening of the trachea wall improved, and she was discharged with a low dose of oral steroids.

However, 5 months after discharge, the patient complained of aggravated dyspnea upon reduction of the oral steroids. She received an increased dose of steroid (methylprednisolone 1 mg/kg/day) with cyclophosphamide in Russia due to an aggravated dyspnea. She was hospitalized again, and bronchoscopy showed a severe SGS with an 80% level (Figure 3C). An emergency tracheotomy was performed, and we discussed additional medical and surgical treatment of the severe SGS. First, carbon dioxide (CO_2_) laser surgery for luminal widening was conducted in four directions of the subglottic area, the front, back, left, and right, via laryngomicroscopic surgery, because the stenosis lesion was close to the vocal cord (approximately 1.5 cm). 

Additionally, intralesional steroid injections were also performed (Figure 3D). Although she received an increased dose of steroid, cyclophosphamide, and intratracheal interventions, she complained of dyspnea again 7 days later, and bronchoscopy showed a 70% level of SGS (Figure 3E). She was transferred to the intensive care unit and mechanical ventilator support was provided due to the risk of respiratory arrest; however, this was ineffective. We discussed the surgery strategy with the thoracic surgery team. As a result of visual inspection in the operating room, a 70% level of SGS was identified immediately under the cricoid cartilage. The area where repetitive narrowing occurred despite several treatments was completely removed, and the resection length was approximately 2 cm. Special attention was paid to maintaining adequate ventilation of the patient during surgery, and paratracheal area dissection was carried out with caution to prevent excessive tension of the trachea resection and anastomosis site. Surgery followed by tracheal cannula removal was successful, and she was discharged with low-dose steroids and cyclophosphamide (Figure 3F). 

**Figure 4:**
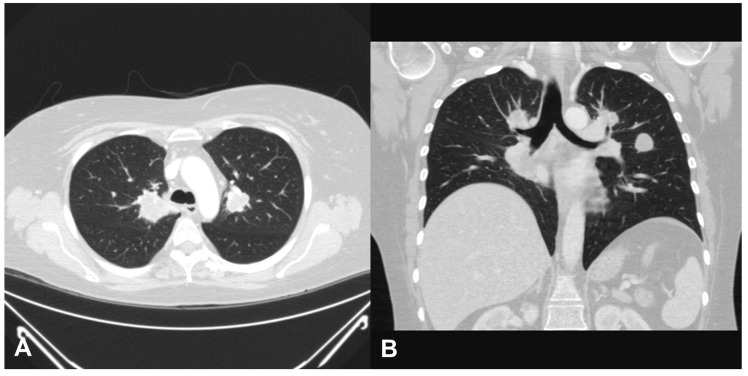
Chest computed tomography scan showed several round or lobular nodules with central necrosis in both lungs 2 years after surgery for subglottic stenosis (**A**, axial view; **B**, coronal view).

Two years later, the patient was admitted for dyspnea again, and a chest CT scan revealed several round or lobular nodules with central necrosis in both lungs (Figure 4A,B). Bronchoscopy revealed no SGS but bronchial obstruction due to necrotic inflammatory change on the right middle lobe. Bronchial biopsy was performed on the right middle lobe. Necrotizing granulomatous inflammation was also observed on histopathological examination (Figure 2B). Owing to frequent recurrence of GPA, rituximab (375 mg/m^2^ of body surface area) was administered with steroids weekly for 4 consecutive weeks, which led to regression of dyspnea, and the dose of steroid was tapered from 60 to 10 mg/day without relapse of SGS after 12 months of follow-up.

## 3. Discussion

GPA is a rare systemic autoimmune disease characterized by systemic granulomatous vasculitis in small to medium-sized vessels [5]. In particular, the upper and lower respiratory tract and kidneys are involved in more than 90% of cases [6]. SGS is defined as narrowing of the airway immediately below the vocal cords with a 16–23% frequency in GPA [7,8]. SGS is more commonly seen in young female patients with GPA. As our patient was a relatively young woman and a saddle nose, we suspected SGS with GPA. However, the recognition of active SGS can be challenging in patients with GPA because it may occur in isolation without evidence of other organ involvement and is misdiagnosed as other respiratory diseases such as asthma, other respiratory infections, or allergies.

The localized or limited forms of GPA phenotypes primarily through the upper respiratory tract, occur to affect younger and more female individuals compared with its diffuse or systemic forms [1,7]. Physicians should consider SGS in young female patients with GPA, particularly in those with severe and destructive sinonasal diseases with no associated renal involvement [1]. In our patient, a transition from localized SGS to pulmonary nodules in diffuse form was observed during the course of GPA. The localized forms are more granulomatous with greater type 1 T helper lymphocyte polarization, while diffuse forms especially present with vascular inflammatory lesions with greater type 2 T helper lymphocyte polarization [9]. Although two phenotypes have been known to have different pathophysiological processes, there is no consensus as to the phenotype definition, treatment, and prognosis.

Rituximab is a monoclonal anti-CD20 antibody and leads to prolonged B-cell depletion, associated with a prolonged clinical response and a fall in ANCA titer [10]. A study reported that rituximab is less effective in treating granulomatous upper airways manifestations of localized GPA than treating generalized systemic diseases [11], which may be due to delayed response of rituximab for localized granulomatous disease. However, rituximab has been used for patients with localized GPA and may be sufficient to induce sustained remission, even among patients with refractory disease and necrotizing granulomatous disease manifestations [12]. In our patient with severe and refractory SGS for more than 6 months, earlier consideration of rituximab may be more effective in treating recurrent SGS.

The presentation of SGS may be independent of the systemic manifestations of GPA and its treatment [3]. The favorable results of the various interventions, such as endoscopic procedures and surgery have been observed regardless of immunosuppressant use (Table 1). Patients with SGS required urgent evaluation and a possibility of tracheostomy for severe cases. About half of SGS patients underwent tracheotomy [13,14], and intratracheal intervention is common (74–80%) [6,7,8]. Endoscopic therapy, such as bronchoscopic dilatation or steroid injection, stent placement, and endoscopic laser surgery, is widely used in SGS [15]. Although limited cases related to the efficacy of radiation therapy have been reported in the literature [16,17], radiation therapy is used in the upper respiratory tract that is refractory to standard therapy [18]. Our patient was treated with systemic immunosuppressants, including systemic steroids and cyclophosphamide, and the initial treatment response was successful, considering the improvement of SGS. However, SGS aggravated and caused significant compromise of airways, which was grade 3 with encircling granulation (71–99% obstruction) of the Myer-Cotton staging system (MCS) [19]. Our patient underwent most endoscopic treatments that we could perform, including steroid injection and laser surgery; however, relapse of SGS occurred, and laryngotracheal resection with reconstruction was enventually performed. Although endoscopic treatment has the advantage of being a less invasive procedure, physicians may consider earlier active surgery in patients presenting with severe and complex airway obstruction because airway reconstruction surgery with the help of decannulation implementation can reduce catastrophic situations such as respiratory arrest.

Recurrent SGS is well-recognized and can occur in 50–75% of patients when GPA is in remission [2,20]. Previous studies have shown variability in ANCA status, concluding that SGS patients are more likely to be C-ANCA/PR3-positive than perinuclear (P)-ANCA/myeloperoxidase-positive [8,14]. In current study, there were no significant differences among ANCA types between patients with and without SGS [21]. Although variations in the ANCA titer are not predictive of relapse, a persistent positive ANCA is predictive of relapse [22]. Our patient revealed positive result of C-ANCA/PR3 at the time of GPA with SGS diagnosis, but all of the ANCA results during each recurrent SGS were negative. The result of anti-PR3 Ab was changed from positive to negative 1 year after laryngotracheal resection with reconstruction. However, the result of anti-PR3 antibody was positive again, which revealed pulmonary lobular nodules with central necrosis in both lungs on chest CT scan. ANCA/PR3 remains controversial as a useful biomarker for the recurrence of SGS.

## 4. Conclusions

It is important to establish an early diagnosis and proper intervention in patients with SGS and GPA because SGS is related to a high risk of morbidities. Early diagnosis of SGS depends on a high degree of suspicion due to nonspecific symptoms, including cough, hoarseness, dyspnea, stridor, and wheezing. Active endoscopic interventions alleviate the symptoms; however, the SGS frequently recurs and develops even with independent systemic immunosuppressants treatment, requiring active surgery in the majority of patients. The management of life-threatening SGS with GPA is complex and requires a multidisciplinary team approach.

## Figures and Tables

**Table 1:** Review of the studies of subglottic stenosis in granulomatosis with polyangiitis.

Author	No.	F (*n*)	Onset Age of SGS (Mean Years)	Medication Alone (*n*)	Endoscopic Procedure			Surgery (*n*)	Tracheostomy (*n*)	Decannulation (*n*)
					Dilatation (*n*)	CS Injection (*n*)	Laser (*n*)			
Girard et al. [20]	26	17	33.1	15	4	2	5	3	0	0
Jordan et al. [23]	9	7	44	0	4	4	4	5	1	0
Taylor et al. [13]	15	9	31.7	0	14	10	15	NR	6	4
Gouveris et al. [24]	4	4	35.6	0	2	3	3	0	0	0
Wolter et al. [25]	8	4	35.6	0	0	2	0	0	1	1
Schokkenbroek et al. [2]	9	8	45.6	0	9	0	1	0	2	1
Nouraei et al. [26]	18	9	40	0	0	31 *	31 *	0	0	0
Gltuh et al. [14]	27	16	40.3	6	NR	11	12	7	11	8
Hoffman et al. [27]	21	16	39.1	NR	64 *	64 *	NR	0	0	0
Horta-Baas et al. [3]	4	3	49.3	0	2	1	0	0	3	2

*, total number of procedure; No., number of patients; F, female; *n*, number of patients; SGS, subglottic stenosis; CS, corticosteroids; NR, not reported.

## Data Availability

The data reported in this paper are available from the medical history of the patient.
